# Human gut microbiome, diet, and mental disorders

**DOI:** 10.1007/s10123-024-00518-6

**Published:** 2024-04-01

**Authors:** Alejandro Borrego-Ruiz, Juan J. Borrego

**Affiliations:** 1 Departamento de Psicología Social y de las Organizaciones, Universidad Nacional de Educación a Distancia (UNED), Madrid, Spain; 2 Departamento de Microbiología, Universidad de Málaga. Instituto de Investigación Biomédica de Málaga y Plataforma en Nanomedicina BIONAND, Málaga, Spain

**Keywords:** Human gut microbiome, Diet, Dietary habits, Mental disorders

## Abstract

Diet is one of the most important external factor shaping the composition and metabolic activities of the gut microbiome. The gut microbiome plays a crucial role in host health, including immune system development, nutrients metabolism, and the synthesis of bioactive molecules. In addition, the gut microbiome has been described as critical for the development of several mental disorders. Nutritional psychiatry is an emerging field of research that may provide a link between diet, microbial function, and brain health. In this study, we have reviewed the influence of different diet types, such as Western, Mediterranean, vegetarian, and ketogenic, on the gut microbiota composition and function, and their implication in various neuropsychiatric and psychological disorders.

## Introduction

For several decades, many dietary patterns have been developed to improve the quality of life and health of people in our society. Dietary patterns constitute the quantity and the variety of foods in a diet, as well as the frequency with which the edibles are ingested. Some of the main dietary variants are (i) high-calorie diets (e.g., Western diet); (ii) mixed-balanced diets (e.g., Mediterranean diet); (iii) plant-based diets (e.g., vegetarian diet); and (iv) low-carbohydrate diets (e.g., ketogenic diet). These diets have different effects on physiological wellness, and some of them share common elements (Clemente-Suárez et al. 2023; Guasch-Ferré and Willett 2021). In spite of that, adherence to a particular dietary pattern depends, ultimately, on geographical, cultural, ethical and environmental awareness, self-image and physical fitness, health maintenance, and psychological well-being (Hargreaves et al. 2023; Tosti et al. 2018; Westman et al. 2003).

The variation of a healthy gut microbiome is considered as a physiological phenomenon that depends on age, ethnicity, lifestyle, and dietary habits (Valdes et al. 2018). According to Gilbert and Lynch (2019), the human-microbial co-dependence is subject to a co-evolution between the human immune system and the nutritional adaptation of the gut microbiome to the available nutrient substrates. The gut constitutes the most important human microbiome, which is not a stable and static system, but a dynamic and functional community that can change in space and time depending on the host physiological factors (Gerber 2014). Although “microbiota” and “microbiome” are often used interchangeably and synonymously, there are some differences between the two terms. The term microbiome encompasses a whole range of microorganisms, including bacteria, archaea, viruses, and fungi, the collection of genomes from all the microorganisms in that environment, as well as microbial structural elements, metabolites, and environmental conditions. The term microbiota is more restricted and describes the group of commensalist, symbiotic, and pathogenic microorganisms found in a given environment (Berg et al. 2020). Numerous studies have shown that the healthy human gut bacteriome in adults consists of 12 bacterial phyla (of which more than 93% belong to Actinomycetota, Bacillota, Bacteroidota, and Pseudomonadota); 18 bacterial families, the most prevalent being *Bacteroidaceae* (65.6%), *Lachnospiraceae* (11.5%), and *Ruminococcaceae* (8.4%); and 59 bacterial genera, with *Bacteroides* as the most abundant (more than 65%) (Almonacid et al. 2019; Costea et al. 2018; Nishijima et al. 2016). In addition, more than 3.8 million of microbial genes have been characterized, a proportion 150 times greater than the entire human genome, of which 99% are of bacterial origin (Li et al. 2014).

The gut-brain axis refers to a two ways communication network that connects the nervous system (central [CNS]; autonomic [ANS]; enteric [ENS]), or the hypothalamic–pituitary–adrenal (HPA) axis, with the intestine via vagal, neuroimmune, and neuroendocrine pathways (Cryan et al. 2019). Intermediate metabolites of the gut microbiome (e.g., short-chain fatty acids [SCFAs] and tryptophan catabolites), cytokines, and neurotransmitters, may be involved in various brain functions (Cryan et al. 2019; O’Mahony et al. 2015; Sandhu et al. 2017). Thus, the gut microbiome also plays a key role in the development of several neuropsychiatric disorders (Borrego-Ruiz and Borrego 2024; Cryan and Dinan 2015; Cryan et al. 2020; Lach et al. 2018).

Dietary patterns are one of the most important determinants of health, exerting their effects through mechanisms such as inflammation, oxidative stress, tryptophan metabolism, epigenetics, CNS and HPA functions, mitochondria, and gut microbiota (Marx et al. 2021; Rinninella et al. 2019). Furthermore, epidemiological studies have shown the involvement of dietary factors in the development of some mental disorders (Jacka et al. 2017; Marx et al. 2021; Ota et al. 2019; Parletta et al. 2019; Sánchez-Villegas et al. 2013), as well as their use as potential therapeutic tools (Adams et al. 2018; Estruch et al. 2018; Jacka et al. 2017). However, only a few studies have established a causal effect of the gut microbiome through dietary factors in mental disorders (Horn et al. 2022).

Based on the influence that diet exerts on gut microbiome composition and function, and also considering the existence of a network of interacting gut-brain communication, it has become clear that the interactions between the three components play a pivotal role in human health. Therefore, in this study, we have reviewed the influence of different diet types, such as traditional dietary habits (Western and Mediterranean diets) and emergent dietary habits (vegetarian and ketogenic diets), on the composition and function of the gut microbiome, and its implication in several mental disorders.

## Method

The present work consists of a narrative review, aimed at collecting and analyzing the existing literature in order to provide a complete and exhaustive overview of the central topic of study. Both authors independently performed a conscientious literature search within the field that matched the topic under investigation. For this purpose, PubMed, Scopus, and Web of Science were examined, between August and September 2023, using different combinations of keywords related to the research object, such as “human gut microbiome”, “diets”, or “mental disorders”. The search strategy also included examining reference list from previous reviews and research papers. Both authors separately evaluated all suitable records, considering studies mostly focused on humans that explored the effect of diet on the gut microbiome and its implication in various neuropsychiatric and psychological disorders. Concerning that, each article found was individually assessed for its pertinence through the screening of title and abstract at first. Duplicate entries were removed, and as well those studies that were not likely to be included in the review due to their subject matters. The full texts of the remaining articles were meticulously inquired, and relevant data from them was extracted for further analysis. In this respect, studies that lacked significant information regarding the relationship between human gut microbiome and diet were excluded from the review, and also those ones that were meta-analyses, based on endocrine disturbances, and related to physiological, self-immune, or viral diseases.

## Dietary types and their influence on the gut microbiome

Diets provide certain nutrients involved in the growth kinetics of gut bacteria, like glycans (inulin, lignin, pectin, cellulose, and fructooligosaccharides), which are indigestible carbohydrates for animals and humans (Cantarel et al. 2012). However, certain gut bacteria, known as primary degraders, which include members of the genera *Bacteroides, Bifidobacterium* and *Ruminococcus*, can break down glycans (Eilam et al. 2014). Moreover, diet can influence the metabolism and immune system of the host, and thus, modulate the shape of the microbiome through various substances, such as indole-derived compounds, vitamins A and D, and polyunsaturated fatty acids (Hibberd et al. 2017; Luthold et al. 2017).

### Traditional dietary habits

Western diet (WD) is characterized by a high content of refined sugars, salt, saturated fats (especially omega-6 fatty acids), and proteins of animal origin (Varlamov 2017). This diet has been related to the occurrence of metabolic and pathological disorders, like type 2 diabetes, obesity, some cancers, and cardiovascular diseases (Clemente-Suárez et al. 2023; Mehta et al. 2017; Piernas et al. 2022).

The gut microbiota profile of individuals fed with a WD is similar to that found in obese individuals (David et al. 2014). A high-fat diet, which contains derived saturated fats, induces a decrease in Bacteroidota levels and an increase in members of both Pseudomonadota and Bacillota phyla in the gut microbiota (Bisanz et al. 2019; Malinowska et al. 2022). Furthermore, WD is rich in animal protein and provokes an increase of *Bacteroides* spp., *Alistipes* spp., and *Bilophila* spp., and a decrease in the levels of *Lactobacillus* spp., *Enterococcus* spp., *Roseburia* spp., and *Eubacterium* spp. (Beam et al. 2021; Muscogiuri et al. 2019; Singh et al. 2017).

The typical WD is associated with chronic inflammation, metabolic syndrome (diabetes, hypertension, and cardiovascular disease), and obesity (Cani 2013; Zinöcker and Lindseth 2018), due to the production of high levels of lipopolysaccharide (LPS) and trimethylamine N-oxide (TMAO) by the altered gut microbiota, and also to the decrease in SCFAs (Singh et al. 2017).

On the other hand, the Mediterranean diet (MD) consists mainly of important sources of fiber (cereals, nuts, legumes, vegetables, and fruits), unsaturated fatty acids, and antioxidant compounds (vitamins, flavonoids, phytosterols, minerals, terpenes, and polyphenols), with moderate consumption of eggs, white meat and fish or seafood, and low consumption of red meat and sweets (Estruch et al. 2018; Nagpal et al. 2019a). Compared to WD, MD has beneficial effects on human health and longevity, reducing the risk of cardiovascular disease, cancer, obesity, and other related metabolic disorders (Herpich et al. 2022; Lopez-Legarrea et al. 2014; Marlow et al. 2013; Muscogiuri et al. 2022; Richardson et al. 2022).

Such dietary adherence leads to a reshaped gut microbiota composition and provokes a higher microbial diversity (De Filippis et al. 2016; Garcia-Mantrana et al. 2018; Mitsou et al. 2017), with an increased population of the bacterial families *Clostridiaceae* and *Lactobacillaceae*, and of the genera *Bacteroides*, *Bifidobacterium*, *Clostridium*, *Faecalibacterium*, *Lactobacillus*, *Oscillospira*, *Prevotella*, and *Roseburia*, and also with a decrease in members of the phyla Pseudomonadota and Bacillota, and the genera *Coprococcus* and *Ruminococcus* (De Filippis et al. 2016; Garcia-Mantrana et al. 2018; Ghosh et al. 2020; Haro et al. 2017; Nagpal et al. 2019a; Pagliai et al. 2020).

### Emergent dietary habits

Vegetarianism consists of a diet free of animal meat, characterized by low-fat and high-fiber components. Although diets based on vegetarianism are considered as emerging diets specially in Western countries, they have been a dietary pattern that dates back to ancient times in diverse cultures, mostly in Eastern geographic environments. There are different variants of vegetarian diets (VD), such as those that include dairy products and eggs (lacto-ovo-vegetarianism); those that include dairy products, but not eggs (lacto-vegetarianism); those that include eggs, but not dairy products (ovo-vegetarianism); and those that are strictly vegan, which do not include any derivative components of animal origin (Parker and Vadiveloo 2019; Xiao et al. 2022).

Several studies have reported a strong association between VD and certain health indicators, such as lower body fat index, lower incidence of type 2 diabetes, lower risk of suffering from cardiovascular disease, lower likelihood of developing various types of cancer, and increased longevity, compared to an omnivorous diet (Craig et al. 2021; Lassale et al. 2015; McMacken and Shah 2017; Olfert et al. 2022; Orlich et al. 2013; 2015; Pawlak 2017).

Adopting a VD reduces β-diversity (the amount of differentiation between species communities) of the gut microbiome, but not the individual diversity at the local scale (α-diversity) (Andermann et al. 2022). Arumugam et al. (2011) found that the prominent enterotypes of the human microbiome are the genera *Bacteroides*, *Ruminococcus*, and *Prevotella*. Different diets alter the distribution of these enterotypes (Wu et al. 2011), leading to an increase in *Prevotella*, *Roseburia*, *Haemophilus*, *Neisseria*, *Aggregatibacter*, and *Veillonella* species, which is associated with long-term adherence to a plant-based diet, although the *Ruminococcus* enterotype is conserved (David et al. 2014; De Filippo et al. 2010; Tomova et al. 2019; Zhang et al. 2018a). More recently, Xiao et al. (2022) reported that vegetarians had increased gut microbiota diversity compared to omnivores, with a higher abundance of the genera *Prevotella*, *Clostridium*, *Lactobacillus*, *Ruminococcus*, *Eubacterium*, and *Faecalibacterium*, and with lower loads of *Bacteroides* and *Bifidobacterium*.

VD has been considered as a healthy and therapeutic dietary pattern for several metabolic and chronic diseases, since it induces a reduction of pathobionts in the intestinal tract (Glick-Bauer and Yeh 2014). Besides, these diets are, in ecological terms, more sustainable than those that include meat, so their negative impact on the planet is significantly smaller (Craig et al. 2021).

The ketogenic diet (KD) is another emergent dietary habit, consisting in a normocaloric, high-fat, and very low-carbohydrate diet, which induces a state of ketosis (Attaye et al. 2021). KD feeding results in increased amounts of acetoacetate, β-hydroxybutyrate, and acetone in the blood and the urine, whose consequences are inhibition of apoptotic proteins, improvement of mitochondrial activity, attenuation of oxidative stress, expression of antioxidant proteins, and modulation of neurotransmitter levels (γ-aminobutyric acid [GABA], glutamate, and monoamines) (Cavaleri and Bashar 2018; Greco et al. 2016; Hartman et al. 2007; Yudkoff et al. 2008). These KD effects provide health benefits by reducing symptoms of affections such as autism, depression, epilepsy, diabetes, and various mental diseases (Bolla et al. 2019; Bostock et al. 2017; Lange et al. 2017; Martin-McGill et al. 2020).

Recent studies have suggested a key role for the gut microbiota in the mechanism of action of the KD (Attaye et al. 2021; Paoli et al. 2019; Rew et al. 2022). Kim et al. (2012) showed, in preclinical studies, that KD can increase inflammation and pro-inflammatory cytokines via the TLR4 signaling pathway. These changes are reflected in the composition of the gut microbiota, with a decrease in members of the Bacillota and an increase in members of the Bacteroidota phyla (Basciani et al. 2020). A study performed on humans, using a modified KD, found a decrease in the abundance of *Bifidobacterium* spp. and *Lachnobacterium* spp. in the gut microbiota; conversely, *Akkermansia* spp., *Slackia* spp., and members of the *Christensenellaceae* family were increased (Nagpal et al. 2019b). In children with severe epilepsy, Lindefeldt et al. (2019) did not found a significant change in the α-diversity of their fecal microbiota. However, a relative abundance of *Bifidobacterium*, *Eubacterium*, and *Dialister* were significantly diminished during the intervention, and an increase in relative abundance of *Escherichia* was also observed in KD-fed children. Another study, in animal and human models, showed that KD feeding decreased the abundance of members of Actinomycetota, *Lactobacillus* spp., and *Bifidobacterium* spp. (Ang et al. 2020). Additionally, these authors concluded that KD could potentially be used as a therapeutic tool to control autoimmune diseases, due to its reduction of pro-inflammatory Th17 cells. Nevertheless, a negative effect of the low carbohydrate diets, like KD, is the reduction in the abundance of the Bifidobacteria group, which has been positively associated with human health (Arboleya et al. 2016).

## Effects of diet on neuropsychiatric and psychological disorders

Food is a source of several bioactive molecules, such as serotonin, dopamine, histamine, GABA, glutamate, and acetylcholine, which have neuroactive properties modulating neural signaling within the ENS, and influencing several brain functions (Briguglio et al. 2018; Burokas et al. 2015; Oriach et al. 2016). Omega-3 fatty acids and various amino acids may influence brain development and function independently of the gut microbiome (Sarris et al. 2015; Wani et al. 2015). Other dietary micronutrients, such as vitamins and minerals, can act as cofactors for enzymes, and be involved in neurotransmitter synthesis, myelination, cellular signaling, and metabolic pathways (Tardy et al. 2020).

### Western diet

WD and obesity are risk factors for neuropsychiatric and psychological disorders, such as mild cognitive impairment, dementia, and depression (Castanon et al. 2014; Pedditzi et al. 2016; Pistell et al. 2010; Xu et al. 2011). WD components could induce neurochemical changes in specific brain regions; in particular, the dysfunction of the hippocampus leads to an impaired cognitive state, disrupting normal intake control and memory tasks (Francis and Stevenson 2013; Kanoski and Davidson 2011; Stevenson et al. 2020).

Long-term exposure to WD could produce addictive eating behaviors through the release of dopamine in the mesocorticolimbic system (Stevenson et al. 2020), and also the dysregulation of the HPA axis through the release of corticosterone, which provokes chronic stress, anxiety, and depression (López-Taboada et al. 2020; Makhathini et al. 2017; Thanarajah et al. 2019). On the other hand, stress triggers changes in the HPA axis that stimulate the production of leptin, ghrelin, insulin, or neuropeptide Y, establishing neuropeptide circuits that regulate intake control, memory, and motivation (Maniam and Morris 2012; Zanchi et al. 2017).

High-fat diets increase the proliferation of adipose tissue phagocytic cells, which leads to the release of pro-inflammatory cytokines and causes a neuroinflammation that is associated with depression, anxiety, and impaired cognitive function (Guillemot-Legris and Muccioli 2017; Kim et al. 2020; Seong et al. 2019). The high levels of endotoxins and the reduced levels of anti-inflammatory gut bacterial species are also linked to neuroinflammation (Noble et al. 2017).

### Mediterranean diet

Numerous studies have shown that adherence to the MD is associated with a better cognition and memory (Lehert et al. 2015), a lower risk of cognitive impairment (Psaltopoulou et al. 2013; Singh et al. 2014; Wu and Sun 2017), a delay in cognitive decline (Chen et al. 2019; Morris et al. 2015), and in the development of neurodegenerative diseases (Karstens et al. 2019; Singh et al. 2014; van den Brink et al. 2019). These beneficial effects of this diet are due to its anti-inflammatory effects and to the increase of SCFAs (Koelman et al. 2022; Koh et al. 2016).

Several authors have pointed out that MD can reduce the risk of depression (Estruch et al. 2018; Huang et al. 2019; Jacka et al. 2017; Lassale et al. 2019), probably due to the abundance of oleic acid, polyphenols, and unsaturated fatty acids that are present in the dietary composition (Bayes et al. 2020; Del Chierico et al. 2014). Although a direct link between polyphenols and an improvement in neurological disorders has not yet been established, Wang et al. (2022) suggest that polyphenols may reverse neurodegenerative and neurocognitive pathologies. In addition, some studies have suggested a lower perceived stress and an improved stress resilience that is associated with the adherence to a MD (Bonaccio et al. 2018). The benefits of MD to improve the development of human health–related quality of life have been confirmed by different interventions in several countries, such as Italy (Bonaccio et al. 2013), Spain (Cabrera-Suárez et al. 2023; Galilea-Zabalza et al. 2018; Martínez-Lapiscina et al. 2013; Valls-Pedret et al. 2015), Greece and the UK (Klonizakis et al. 2019), Greece (Trichopoulou et al. 2015), Australia (Jacka et al. 2017), and the USA (Bhushan et al. 2018; Gigic et al. 2018).

### Vegetarian diet

There are conflicting results regarding the influence of the VD on the incidence of depression; while some studies have found a positive association (Dobersek et al. 2021; Fazelian et al. 2022; Forestell and Nezlek 2018; Hibbeln et al. 2018; Kohl et al. 2023; Lavallee et al. 2019; Li et al. 2019; Matta et al. 2018; Stokes et al. 2011), others concluded that the VDs are not linked to depression (Beezhold and Johnston 2012; Beezhold et al. 2010; Jin et al. 2021; Michalak et al. 2012; Northstone et al. 2018; Norwood et al. 2019; Storz and Ronco 2023; Timko et al. 2012). It has been suggested that deficiencies of essential amino acids, such as methionine, tryptophan, and tyrosine, which are present at very low concentration in plant-based diets, may explain the relationship between these diets and the processes of depression (Schmidt et al. 2016), based on the metabolism of dopamine and serotonin (Aucoin et al. 2018).

### Ketogenic diet

A large body of literature has reported that KD significantly reduces epileptic seizures, suggesting it as a therapeutic tool (Kossoff et al. 2018; Olson et al. 2018; Xie et al. 2017; Zhang et al. 2018b). KD helps to rebalance neurotransmitter systems, stabilizes neural networks, and improves neuroplasticity (Brietzke et al. 2018; Mujica-Parodi et al. 2020). Moreover, Campbell and Campbell (2020) suggested that KD may help to reduce the symptoms of bipolar disorder by bypassing the reduction of the mitochondrial defects.

The KD can also potentially cause cognitive impairment and alter the gut microbiota (increase of *Bilophila wadsworthia*). These facts disrupt hippocampal synaptic plasticity, neurogenesis, and gene expression (Olson et al. 2021). Shegelman et al. (2021), studying the effects of a KD in adult patients with chronic epilepsy, reported that this diet may have a beneficial effect on mental condition, reducing anxiety and depression. In a retrospective study, Danan et al. (2022) showed significant improvements in depression and in psychotic symptoms in individuals with severe mental disorders subjected to a KD. Similarly, Adams et al. (2022) reported an important enhancement in the mood of outpatients with type 2 diabetes that were treated with a KD for 2 years. However, Iacovides et al. (2019) found no significant differences in mood, cognitive performance, or subjective sleep quality, between the two groups of individuals (KD subjects and isocaloric high-carbohydrate low-fat diet subjects).

## Diet, microbiome, and mental disorders

Dietary types change the composition and structure of the gut microbiome since birth with the introduction of solid foods in feeding, and with the aging (Claesson et al. 2012; Laursen et al. 2017). The gut microbiome of vaginally delivered infants is similar to the vaginal microbiota of the mother, while infants delivered by C-section acquired microbial communities found on human skin and opportunistic pathogens commonly associated with the healthcare environment (Dominguez-Bello et al. 2010) (Table 1). Breastfeeding provides a mixture of (i) nutrients that induce bacterial growth; (ii) antimicrobial agents; (iii) secreted-IgA that promotes a regulatory immune system; and (iv) human milk oligosaccharides, considered as prebiotics, that promotes the growth and function of beneficial microorganisms (O'Sullivan et al. 2015). The method of infant feeding by breastfeeding in the first 3 months of life shows a change in the microbial composition of the gut microbiome, with an increase in the abundance of the genera *Bifidobacterium*, *Corynebacterium*, *Enterococcus*, *Lactobacillus*, *Propionibacterium*, *Sneathia*, and *Streptococcus* (Table 1), and a decrease in the abundance of the genera *Bacteroides* and *Staphylococcus*. However, formula-fed infants have a different microbial composition, with the most prevalent bacterial genera being *Atopobium* (phylum Actinomycetota), *Clostridium*, *Enterococcus*, *Lactobacillus*, and *Granulicatella* (phylum Bacillota), *Bacteroides* (phylum Bacteroidota), *Citrobacter*, *Enterobacter*, and *Escherichia* (phylum Pseudomonadota), and *Bilophila* (phylum Thermodesulfobacteriota) (Cortes-Macías et al. 2021; Yao et al. 2021). During weaning, a variety of solid foods and new nutrients are introduced, and the microbial α-diversity and pH of the gut microbiome increase, resulting in the replacement of Actinomycetota and Pseudomonadota by Bacillota and Bacteroidota phyla as the dominant members of the infant gut microbiome (Koenig et al. 2011). In addition, between 9 and 18 months of age, a replacement of the dominant bacterial genera occur, and a difference was found in the gut microbiome of infants who were no longer breastfed compared to those who were breastfed longer; in the former, the most predominant genera are *Akkermansia*, *Bacteroides*, *Bifidobacterium*, *Bilophila*, and the members of the phylum Bacillota: *Anaerostipes*, *Blautia*, *Clostridium*, *Faecalibacterium*, *Roseburi*a, and *Ruminococcus*; whereas in the longer breastfed the most abundant bacterial genera are *Collinsella*, and the members of the Bacillota phylum: *Lactobacillus*, *Megasphaera*, and *Veillonella* (Bäckhed et al. 2015; Vallès et al. 2014). These microbial changes are associated with the increased protein intake (members of *Lachnospiraceae*), dietary fiber intake (members of *Prevotellacea*), and increase in mucin production (genus *Akkermansia*) (Milani et al. 2017). The transition from an exclusively milk-based diet to solid foods induces the development of a mature microbiota containing genes responsible for degradation of complex carbohydrate and xenobiotic compounds, as well as those of vitamin production (Koenig et al. 2011). Thereafter, the microbiota remains unstable, suffering sudden microbial succession phenomena, until the infant is 2 to 3 years old, when the microbiota reaches a composition similar to that of adults (Yatsunenko et al. 2012).

**Table 1:** Influence of feeding on the gut microbiome composition in early life

Neonates	Prevalent bacterial phyla	Dominant bacterial genera
Vaginally born^a^	Actinomycetota	*Bifidobacterium*
		*Collinsella*
	Bacillota	*Clostridium*
		*Lactobacillus*
		*Streptococcus*
		*Veillonella*
	Bacteroidota	*Bacteroides*
		*Parabacteroides*
		*Prevotella*
	Fusobacteriota	*Sneathia*
	Pseudomonadota	*Escherichia*
	Verrucomicrobiota	*Akkermansia*
C-section born^a^	Actinomycetota	*Corynebacterium*
		*Propionibacterium*
		*Slackia*
	Bacillota	*Staphylococcus*
		*Streptococcus*
		*Veillonella*
	Pseudomonadota	*Enterobacter*
		*Haemophilus*
Breastfeeding^b^	Actinomycetota	*Bifidobacterium*
		*Corynebacterium*
		*Propionibacterium*
	Bacillota	*Enterococcus*
		*Lactobacillus*
		*Streptococcus*
	Fusobacteriota	*Sneathia*
Formula-fed^b^	Actinomycetota	*Atopobium*
	Bacillota	*Clostridium*
		*Enterococcus*
		*Granulicatella*
		*Lactobacillus*
	Pseudomonadota	*Citrobacter*
		*Enterobacter*
		*Escherichia*
	Thermodesulfobacteriota	*Bilophila*
Intake of solid foods^c^	Actinomycetota	*Bifidobacterium*
		*Collinsella*
	Bacillota	*Anaerostipes*
		*Blautia*
		*Clostridium*
		*Faecalibacterium*
		*Lactobacillus*
		*Megasphaera*
		*Roseburia*
		*Ruminococcus*
		*Veillonella*
	Bacteroidota	*Bacteroides*
	Thermodesulfobacteriota	*Bilophila*
	Verrucomicrobiota	*Akkermansia*

^a^According to Nuriel-Ohayon et al. (2016) and Yao et al. (2021)

^b^According to Cortes-Macías et al. (2021) and Yao et al. (2021)

^c^According to Bäckhed et al. (2015) and Vallès et al. (2014)

These strong shifts in the gut microbiota profile have their consequences on several mental functions (David et al. 2014; Yatsunenko et al. 2012), through the secretion of biologically active compounds, including SCFAs, tryptophan catabolites, polyamines, and histamine (Lach et al. 2018; Lukić et al. 2022; Silva et al. 2020; Sudo 2019). The most quantitatively important metabolites are SCFAs that are produced by the microbial degradation of indigestible dietary fibers, proteins, and glycoproteins (Wong et al. 2006). SCFAs, such as butyrate, acetate, and propionate, can act as signaling molecules that locally modulate the gut function from the duodenum to the colon; and through enteroendocrine cells they can also control liver, muscle, and brain metabolism, thereby influencing the host energy homeostasis (Dalile et al. 2019; den Besten et al. 2013; Silva et al. 2020). In addition, SCFAs have neuroactive properties through the induction of neuroinflammatory responses (Dalile et al. 2019). Other biologically active compounds are indole and 5-hydroxytryptamine, derived from tryptophan metabolism, which regulate the secretion of the serotonin and melatonin (Lukić et al. 2022), and may serve as signaling molecules for intercellular communication between microorganisms and host cells (Lee and Lee 2010).

Tables 2 and 3 show the relationship between diet, the shape of the gut microbiome, its metabolomes, and mental disorders. WD induces a shift in the gut microbiome composition, which induces the synthesis of microbial metabolites and soluble substances that affect brain functions through the gut microbiota–brain axis, increasing the symptoms of schizophrenia and dementia, as well as the effects on several psychological features, such as cognitive impairment, stress, depression, and anxiety (Aucoin et al. 2018; Jacka et al. 2017; López-Taboada et al. 2020) (Table 2). On the contrary, MD promotes the production of bacterial intermediates that reduce anxiety, depression, and symptoms of bipolar disorder and schizophrenia, and also the increase of stress resilience (Bonaccio et al. 2013; Dinu et al. 2022; Madani et al. 2022) (Table 2).

**Table 2:** Relationship between traditional dietary habits, gut microbiome composition, microbial metabolites, and mental disorders

Parameters	Western	Diet	Mediterranean	Diet
	Increase	Decrease	Increase	Decrease
Bacterial genera	*Escherichia*	*Bacillus*	*Clostridium*	*Coprococcus*
	*Klebsiella*	*Enterococcus*	*Faecalibacterium*	*Ruminococcus*
	*Alistipes*	*Eubacterium*	*Lactobacillus*	*Escherichia*
	*Bacteroides*	*Lactobacillus*	*Oscillospira*	
	*Bilophila*	*Roseburia*	*Roseburia*	
		*Prevotella*	*Bacteroides*	
			*Prevotella*	
			*Bifidobacterium*	
Microbial metabolites	Pro-inflammatory cytokines	SCFA^a^	SCFA	p-cresol sulfate
	TMAO^b^		Anti-inflammatory interleukin 10	Indoxyl sulfate
	LPS^c^		GABA^d^	
Biological effects	Neuroinflammation			Inflammation
	Food intake			Oxidative stress
	Fat deposition			
Brain functions	Cognitive impairment		Insulin resistance	Anxiety
	Dementia symptoms		Stress resilience	Depression
	Schizophrenia symptoms			Bipolar disorder symptoms
	Stress			Schizophrenia symptoms
	Depression			
	Anxiety			

^a^Short-chain fatty acids; ^b^Trimethylamine N-oxide; ^c^Lipopolysaccharide; ^d^*γ*-aminobutyric acid

**Table 3:** Relationship between emerging dietary habits, gut microbiome composition, microbial metabolites, and mental disorders

Parameters	Vegetarian	Diet	Ketogenic	Diet
	Increase	Decrease	Increase	Decrease
Bacterial genera	*Clostridium* *Eubacterium* *Faecalibacterium* *Lactobacillus* *Roseburia* *Ruminococcus* *Veillonella* *Aggregatibacter* *Haemophilus* *Neisseria * *Prevotella*	*Bacteroides* *Bifidobacterium*	*Escherichia* *Sutterella* *Bacteroides* *Parabacteroides* *Slackia* *Bilophila* *Akkermansia*	*Dialister* *Eubacterium* *Faecalibacterium* *Lachnobacterium* *Roseburia* *Ruminococcus* *Turicibacter* *Bifidobacterium* *Desulfovibrio*
Microbial metabolites	Polyamines Retinoic acids SCFA	5-hydroxytryptamine TMAO LPS GABA	Ketone bodies	5-hydroxytryptamine SCFA
Biological effects	Dopamine synthesis	Inflammation Melatonin synthesis Serotonin synthesis Alzheimer’s disease symptoms	Inflammation Depression	Neuroinflammation Melatonin synthesis Serotonin synthesis Amyloid deposition
Brain functions	Affective disorders			
	Depression	Anxiety Cognitive impairment		ASD symptoms^a^ Seizure symptoms Sleep disorders

^a^Autism spectrum disorder

Regarding VD, contradictory results were observed, since SCFAs, polyamines from protein metabolism, and retinoic acids, reduce anxiety, depression, cognitive impairment, and Alzheimer’s symptoms; whereas other intermediates, such as GABA, TMAO, LPS, and 5-hydroxytryptamine, increase affective disorders and depression (Iguacel et al. 2021; Jain et al. 2022) (Table 3). KD induces the synthesis of ketone bodies and suppresses the release of SCFAs and 5-hydroxytryptamine. These intermediates increase depression, but reduce epileptic seizure symptoms, amyloid plaque deposition, and autism symptoms (Grigolon et al. 2020; Shegelman et al. 2021; Tillery et al. 2021) (Table 3).

## Future perspectives and conclusions

Diet is the most important environmental factor influencing the composition and shape of the gut microbiome. This microbiome plays a critical role in host health, including the development of the immune system, the metabolism of nutrients, and the synthesis of bioactive molecules. The mechanisms of action and the extent to which bacterial metabolites may influence the brain function are poorly understood, due to the complexity of the pathways involved in the gut–brain axis; however, this deficiency could be addressed with new advances in metabolomic technology. In this sense, new sequencing technologies will allow us to gain a deeper insight into the composition of the microbiota and its association with neuropsychiatric and psychological disorders.

In recent years, a growing body of research has been focused in the interplay between dietary patterns, gut microbiota composition, and their collective influence on mental health (Taylor et al. 2019). Thus, the role of diet in the etiology and treatment of neuropsychiatric disorders has been proposed (Sarris et al. 2015). Nevertheless, the influence of diet on brain health is a complex and multifactorial-dependent process. Therefore, how nutrition affects gut microbiota composition, and its consequences in psychiatric and psychological disorders, need to be clarified in future by the improvement of human clinical interventions. Further research in this area, then, will elucidate the modulating effects of dietary components on the intestinal microbiota, as well as their potential impact on psychological well-being. Although different types of diets have already been studied with that purpose, it could be pertinent to assess the divergent impacts of dietary variations under the general diet categorization. In this regard, especially the variants of plant-based diets should not be examined together, given the significant difference between them with respect to the presence of certain foods.

Two types of intervention, probiotic and dietary, have been proposed to link gut microbiota, behavioral changes, and diet. Several psychobiotics added as dietary supplements may improve brain functioning and act as therapeutic tools for neuropsychiatric disorders (Dinan et al. 2013). Prebiotic and dietary interventions, including high-fat foods and polyphenols, may be feasible as long-term interventions to reduce certain mental disorders. The use of aliments with a high content of polyamines, as well as the use of probiotics that influence gut bacteria to synthesize polyamines, could be promising approaches for the prevention of cognitive impairment, including Alzheimer’s disease (Hirano et al. 2021; Valdes et al. 2018; Wang et al. 2022). In this regard, new strategies would be based on the development of probiotic treatment with newer bacteria, on the combination of probiotics and prebiotics (synbiotics), and on personalized nutritional interventions (Chua et al. 2017; Zamroziewicz and Barbey 2016; Zeevi et al. 2015). The development of predictive nutrient patterns will provide empirical nutrition therapies for the treatment of cognitive and neurological impairments in the brain.

Therefore, future methodological advances in nutritional epidemiology are needed, through the development of biochemical markers of dietary intake, studies of holistic dietary patterns, and the application of nutrient biomarker patterns (NBP). The use of the NBP method has revealed various nutrient patterns that influence cognitive impairment and brain disorders (Bowman et al. 2012). Metabolomics could provide a tool to investigate the interactions between dietary components and mental function, by characterizing individual dietary phenotypes and elucidating the mechanisms of health status. However, the validation of dietary markers, and the identification of specific patterns of the food metabolome associated with a healthy brain, are still unknown (Scalbert et al. 2014).

Nutritional psychiatry is a relatively new field of research that has evolved from preclinical outcomes and a series of cross-sectional and epidemiological studies linking diet to various aspects of mental health, as well as from the insights of microbiome science, which have provided a relationship between diet, microbial function, and brain health (Adan et al. 2019; Teasdale et al. 2020). The potential future implications of nutritional psychiatry will include the role of diagnostic testing of the gut microbiome to identify targets for personalized psychological and psychiatric treatments, and the potential for integrative approaches combining dietary interventions, pharmacotherapy, and cognitive-behavioral treatments.

In conclusion, the gut microbiome-brain network connects three compartments: the brain, the gut, and the microbiome. All of them are linked by bidirectional connections with multiple feedback loops, creating a non-linear system. Different dietary components influence the brain, the gut, and the gut microbiome through different communication channels. Dietary components can directly affect the gut and reach the brain after absorption in the small intestine. Diet can also influence the composition and diversity of the gut microbiota and the microbial metabolism, thereby modulating the gut connectome. Some of the microbial-derived molecules are absorbed and reach the brain via the systemic circulation and/or the vagus nerve. Furthermore, the brain can modulate the microbiome directly through the action of neuroactive substances released into the gut lumen that affect microbial gene expression, or indirectly by altering the environment of the gut microbiome. On the other hand, there is a need for clinical evidence, based on randomized controlled trials, and prebiotics, probiotics or fecal microbiota transplantation, to assess dietary changes in gut microbiota composition and their influence on psychological outcomes.

## Data Availability

No datasets were generated or analysed during the current study.
